# A Simple View on the Interval and Fuzzy Portfolio Selection Problems

**DOI:** 10.3390/e22090932

**Published:** 2020-08-25

**Authors:** Krzysztof Kaczmarek, Ludmila Dymova, Pavel Sevastjanov

**Affiliations:** Department of Computer Science, Czestochowa University of Technology, Dabrowskiego 73, 42-201 Czestochowa, Poland; krzysztof.kaczmarek@icis.pcz.pl (K.K.); dymowa@icis.pcz.pl (L.D.)

**Keywords:** portfolio selection, risk aversion criterion, profit criterion, interval analysis, fuzzy sets, multiple criteria optimization

## Abstract

In this paper, first we show that the variance used in the Markowitz’s mean-variance model for the portfolio selection with its numerous modifications often does not properly present the risk of portfolio. Therefore, we propose another treating of portfolio risk as the measure of possibility to earn unacceptable low profits of portfolio and a simple mathematical formalization of this measure. In a similar way, we treat the criterion of portfolio’s return maximization as the measure of possibility to get a maximal profit. As the result, we formulate the portfolio selection problem as a bicriteria optimization task. Then, we study the properties of the developed approach using critical examples of portfolios with interval and fuzzy valued returns. The α-cuts representation of fuzzy returns was used. To validate the proposed method, we compare the results we got using it with those obtained with the use of fuzzy versions of seven widely reputed methods for portfolio selection. As in our approach we deal with the bicriteria task, the three most popular methods for local criteria aggregation are compared using the known example of fuzzy portfolio consist of five assets. It is shown that the results we got using our approach to the interval and fuzzy portfolio selection reflect better the essence of this task than those obtained by widely reputed traditional methods for portfolio selection in the fuzzy setting.

## 1. Introduction

The mean-variance (*M*–*V*) model developed by Markowitz (1952) [1] made a great contribution to the portfolio selection theory, considering a return as the mean and a risk as the variance. For example, in [2], the standard portfolio selection of mean-variance model in its bi-objective form and probabilistic setting is presented as a bi-objective quadratic programming problem under cardinality and quantity constraints. As this problem is NP-hard, a new effective iterative method for it solving is developed. The variance was treated as a portfolio risk.

The initial (*M*–*V*) model was improved by different researchers introducing different measures of risk, for example, mean-semivariance models [3,4], mean absolute deviation model [5], mean semi-absolute deviation models [6,7], mean absolute deviation skewness model [8], etc.

The above models are based on the probabilistic treatment of uncertainty: the returns of assets are considered as random variables with corresponding probability distributions. However, in practical applications, often it is hard or even impossible to obtain such probability distributions with acceptable accuracy. This is the consequence of many objective and human factors affected the complex modern financial markets. However, an appearance in 1965 of the theory of fuzzy sets developed by L.A. Zadeh [9] made it possible to take into account these factors and operate with other kinds of information in the formulation and solution of portfolio selection problems.

Therefore, a growing interest in the formulation and solution of portfolio selection problems in the fuzzy setting has being observed during the last two decades. For instance, in [10], a possibilistic approach to the fuzzy portfolio selection with highest utility score is used. In [11], the fuzzy portfolio selection model is used in investment. The problems of fuzzy portfolio selection with the use of possibility theory are analyzed in [12]. In [13,14], the credibility theory is used for the solution of fuzzy portfolio selection problems. The portfolio analysis is used in [15] for the formulation and implementation of strategic decision-making model in the fuzzy setting.

A multiobjective fuzzy mean-semivariance-entropy model for portfolio selection was developed in [16]. It simultaneously optimizes the return, risk, and portfolio diversification, taking into account transaction costs, liquidity, buy-in thresholds, and cardinality constraints. The possibilistic semivariance is used to measure risk. A fuzzy multiobjective approach for portfolio selection is proposed in [17], which makes it possible to consider not only return and downside risk criteria, but also to include environmental, social, and governance (ESG) scores in the investment decision-making process. It is noted in this paper that the mean-absolute semi-deviation of returns is a more appropriate risk measure than variance of returns, as it focuses exclusively on adverse deviations. The propositions made in [18] to extend the mean-semivariance portfolio selection model to a multiobjective credibilistic model that besides the risk and return, also consider the price-to-earnings ratio to measure portfolio performance. Uncertain future returns and PER ratio of each asset are approximated using L-R power fuzzy numbers. It is important that the used semivariance is a better risk measure than the classical Markowitz’s variance as it only deals with adverse deviations. On the other hand, semivariance, being the modified form of variance, cannot be free of all discussed in the literature drawbacks of variance treated as a risk. To capture the coherence of the investor’s expectation in [19], new trapezoidal fuzzy numbers with an adaptive index were proposed. Using these fuzzy numbers, the membership degrees for favorable and unfavorable scenarios are transformed consistently to avoid the logical confusion. The possibilistic expected mean, variance, and skewness were modified in the framework of proposed approach. The introduced new trapezoidal fuzzy numbers were used in the fuzzy mean-variance model and mean-variance-skewness model for the optimal asset allocation. In [20], a multiobjective fuzzy portfolio selection approach, where possibility distributions are given by fuzzy numbers from the information supplied by the decision-making environment (investor, analyst, financial market environment, etc.) is proposed. Moreover, the investor’s preferences were explicitly incorporated through the concept of satisfaction functions. In general, the aim of the paper is to find the best compromise between several objectives (return, risk, and liquidity of portfolio) in fuzzy environment, based on improvements and the fuzzy extension of the Markowitz’s mean-variance model. In [21,22], the fuzzy portfolio selection problems were studied in the case of multiple-decision objectives. A comprehensive model for the fuzzy multiobjective portfolio selection is proposed in [23]. It is based on the synthesis of fuzzy mean-semivariance model and DEA cross-efficiency model. In [24], two multiobjective fuzzy portfolio selection models with variance and conditional value at risk (CVaR) as risk measures, respectively, along with the objectives of liquidity and entropy were proposed. The inherent uncertainty of the investment market was incorporated through trapezoidal fuzzy returns, which were handled using the credibility theory. In addition to the usual realistic constraints of lower and upper bounds on investment in an asset, the capital budget, and no short selling, both the models were also constrained by a minimum return threshold constraint. These multiple objectives of both the portfolio models were then aggregated using the weighted sum approach. Random sample portfolios of different sizes were generated in accordance with the feasibility constraints of the model. The efficiencies of these portfolios were then evaluated with multiple inputs (risk and entropy) and multiple outputs (return and liquidity) using DEA. Furthermore, the inefficient portfolios were rebalanced using an existing DEA improvement technique to offer investors more choices of efficient portfolios. It should be emphasized that modified variance was used as inherent part of portfolio risk in the conditional value at risk model as well. In [25], J.Y. Campbell indicates that the standard mean-variance model does not perform well in explaining household investment behavior in practice. It is worthwhile to note that the results of the standard mean-variance model are based on the assumption that investors face only portfolio risk when making portfolio selection decisions. Yet, in reality investors often face other sources of risk linking variations in labor income, proprietary income, income from real estate, and unexpected expenses related to health issues. These sources of risk are referred to as background risk. Therefore, in [26] the studies were carried out to show how background risk affects individual investment decisions under the framework of uncertainty theory. An uncertain mean-variance model gives its optimal solution when the returns of stocks and background asset obey normal uncertainty distributions. On this basis, the authors studied the characteristic of the mean-variance efficient frontier of the stock portfolio in the presence of background risk. In this case, it seems somewhat strange that the modified mean-variance model based on the treating of variance as a risk measure is used in the situation when according to Campbell (background risk exists) such models cannot work well. Relative recently, some papers devoted to the multiple-period (dynamic) fuzzy portfolio selection problems were published. The fuzzy multiple-period portfolio selection model with different rates of borrowing and lending was presented in [27]. In [28], the authors proposed several multiple-period fuzzy portfolio selection models considering multiple decision criteria. In [29], a possibilistic mean-variance model for multiple-period fuzzy portfolio selection is presented and analyzed. A multiple-period fuzzy portfolio selection model formulated with the demand on return and the constrained risk is proposed in [30]. In [31], a multiple-period fuzzy portfolio optimization problem with minimum transaction costs is analyzed and discussed. A fuzzy multiple-criteria multiple-period portfolio selection problem based on the proposed credibilistic mean-entropy model is presented in [32]. In [33], a numerical integral-based simulation algorithm (NISA) is proposed to approximate the expected value, variance, and skewness of fuzzy numbers. A multiple-period multiple-criteria portfolio selection problem is formulated and solved using a genetic algorithm. A credibility-based mean-semi-entropy multiple-period portfolio model, considering background risk and several constraints, namely, cardinality, liquidity, and buy-in thresholds is formulated and solved in [34]. In [35], the multi-period portfolio selection problem was formulated as a bi-objective optimization model taking into account the transaction cost and bankruptcy of investor. The model was presented in the uncertainty setting (in the sense of Uncertainty theory). The most important criteria were the modified portfolio return and the risk treated as modified variance. In [36], with the use of possibility theory, a new multiple-objective portfolio selection model with discounted transaction costs is developed. To take into account the relative importance and the mutual conditionality of local criteria, a weighted max–min fuzzy goal programming approach is introduced and applied.

In many real-world situations, we know with acceptable reliability only the ranges of possible values of asset’s future returns. Therefore, we should deal with interval type of uncertainty. Although this type of uncertainty is the simplest one and commonly occurring in practice, we have found in the literature relatively few papers devoted to the portfolio selection problems in the interval setting. A portfolio selection model based on three interval-valued local criteria—return, risk, and liquidity—is proposed in [37]. In [38], an interval programming portfolio selection model based on the interval-valued expected return and the interval-valued covariance is formulated and implemented. The problems of multiple-criteria interval portfolio selection were studied in [39,40]. In [41], the prices of stocks are treated as interval-valued variables. In [42], the classical mean-variance portfolio selection model was transformed to the more general mean-variance-skewness one with interval-valued transaction costs. The possible effects of the decision interval on the stock’s share in optimal portfolios was investigated in [43]. In [44], a class of possibilistic portfolio selection models with interval coefficients was analyzed with its application. An interval-valued version of the mean-absolute deviation portfolio selection optimization problem was considered in [45]. In [46], a dynamic (multiple-period) interval portfolio selection model with interval-valued returns, risks, and transaction costs was proposed. In [47], considering the security returns with interval expected returns as uncertain variables, a mean-semi absolute deviation model within the framework of uncertainty theory was developed.

Based on the analysis of cited above papers, we can say that the use of fuzzy and (in relevant cases) interval representation of uncertain information available allows us to avoid some limitations of classical probabilistic approach to the portfolio selection concerned mainly with non-symmetrical distributions of asset returns that we usually meet in practical investment. The modern portfolio selection theory is based on different modifications of classical mean-variance model. Generally, the portfolio selection is a multiple-criteria task and can be formulated using different sets of local criteria. Nevertheless, two main local criteria—maximization of portfolio return and risk minimization—are always presented in different forms dependent on the type of uncertainty dominating in the considered problem. While the mean of corresponding probabilistic or fuzzy distribution of the portfolio return is usually treated as the return maximization criterion without doubts, the use of variance as the measure of the portfolio risk is not so obvious. Therefore, as an alternative to the variance in the portfolio risk assessment, an entropy (probabilistic or fuzzy) of portfolio return distribution is increasingly applied. Nevertheless, it can be seen that the approaches to estimation of portfolio risk based on the variance and the entropy are very close from the methodological point of view.

However, the mean-variance (MV) model has some drawbacks and limitations [24]. One such a drawback is that it considers high returns as equally undesirable as low returns, i.e., it disregards the asymmetry of probability distributions [48]. Another limitation is the incompatibility of the MV model with the axiomatic models of preferences under risk [49]. Moreover, it imparts little information about the loss investors may have to bear, while the loss is investors’ prime concern. Despite all these limitations, variance is still widely used as a benchmark for measuring a risk in a portfolio. Consequently, several researchers and practitioners have explored various risk measures that can be used to segregate desirable upside movements from undesirable downside movements. Among those risk measures, value at risk (VaR) is one such widely accepted popular risk measure. The VaR of an investment is the possibility of the utmost loss with a known confidence level. However, VaR fails to provide any information regarding the losses exceeding it, and it also does not obey the coherence axioms of homogeneity, sub-additivity, monotonicity, and translational invariance. To resolve these inherent inadequacies in VaR, Rockafeller and Uryasev [50] proposed the conditional value at risk (CVaR), which is given as “the weighted average of VaR and the losses exceeding it”. Consequently, CVaR has been widely applied to manage risk in portfolio optimization problems [51,52,53]. However, in financial modeling, a debate is almost always going on about VaR versus CVaR for efficient risk management. Both VaR and CVaR are to some extent based on statistical methods which need relatively strong demands concerned with input data.

There is a field of portfolio management where the achievements of conventional portfolio selection theory practically are not used or used in some small extent.

Therefore, nowadays, the growing interest is observed in the use of stock trading systems for portfolio management [54,55,56]. This approach seems to be very fruitful as it is very close to the investment practice, but the concepts of risk management in such systems are relatively far from those in conventional portfolio selection. Nevertheless, this is out of scope of the current paper. In our opinion, the core of problem lies, implicitly, in the lack of commonly accepted meaningful verbal formulation of what is the portfolio risk.

Therefore, in the current paper we propose a simple view on the portfolio selection problem, which makes it possible to introduce an another method of treating portfolio risk as the measure of possibility to earn unacceptable low returns of portfolio and formulate a simple mathematical formalization of this measure. In a similar way, we treat the criterion of portfolio’s return maximization as the measure of possibility to get a maximal return. As the result, we formulate the portfolio selection problem as a multiple-criteria optimization task. Then, we study the properties of the developed approach using critical examples of portfolios with interval and fuzzy valued returns. The α-cut representation of fuzzy returns is used. To validate the proposed method, we compare the results we obtained using it with those obtained with the use of fuzzy versions of seven widely reputed methods for portfolio selection. As in our approach we deal with the multiple-criteria task, the three most popular methods for the local criteria aggregation are compared using the known example of portfolio consist of five assets. It is shown that the results we got using our approach to fuzzy portfolio selection reflects better the essence of this task than those obtained by widely reputed traditional methods of portfolio selection in the fuzzy setting.

This paper is organized as follows. In Section 2, using the examples of interval-valued portfolio return, we show that the treating of variance as the portfolio risk measure may provide unacceptable counterintuition results. Then, based on the introduced simple view on the portfolio selection problem, we present the new concepts of risk minimization and return maximization criteria. Using these criteria and the three most popular methods of local criteria aggregation, the bicriteria interval-valued portfolio selection task is developed and implemented. Based on the number of illustrative examples, we have shown that a proposed new approach to the interval-valued portfolio selection provides results that coincide with the investor’s intuition and common sense. Section 3 presents a fuzzy extension of proposed in Section 2 approach to the interval-valued portfolio selection. This extension is based on the α-cut representation of fuzzy returns. Using some numerical examples we have shown that properties of proposed approach are logically validate and reflect well an essence of fuzzy portfolio selection. To validate our approach, we compare the results we obtained using it with those obtained with the use of fuzzy extensions of seven widely reputed methods for portfolio selection. As in our approach we deal with bicriteria task, the three most popular methods for local criteria aggregation are compared using the known example of portfolio consist of five assets. It is shown that the results we got using our approach to fuzzy portfolio selection reflect better the essence of this task than those obtained by widely reputed traditional methods of portfolio selection in the fuzzy setting. Section 4 concludes with some remarks.

## 2. An Interval-Valued Portfolio Selection Based on a Simple View on the Local Criteria of Portfolio Quality

Here, we start from the consideration of interval-valued portfolio selection problem. This is not an abstract simplification of reality as in practice often only the ranges (intervals) of future values of the asset returns are known to the investor with an acceptable reliability. On the other hand, in Section 3, a direct fuzzy extension of interval-valued portfolio selection task with the use of α-cut representation of fuzzy returns will be proposed and analyzed. Moreover, it is worthy to not here that a crisp interval is the asymptotic case of trapezoidal fuzzy value when its support is equal to its core.

Therefore, let $ar_{i}$ and $as_{i}$, and *i* = 1 to *N*, be asset returns and asset shares, respectively, of the portfolio consist of *N* assets such that $ar_{i}$ = $\left[\underline{ar_{i}},\bar{ar_{i}}\right]$ are intervals and $as_{i}$ are real values such that $\sum_{i=1}^{N}as_{i}=1$.

Then, the overall interval portfolio return is calculated as follows,

(1) $$OPR=\sum_{i=1}^{N}ar_{i}as_{i}=\left[\underline{OPR},\;\bar{OPR}\right].$$

Let us consider the critical example.

**Figure 1:**
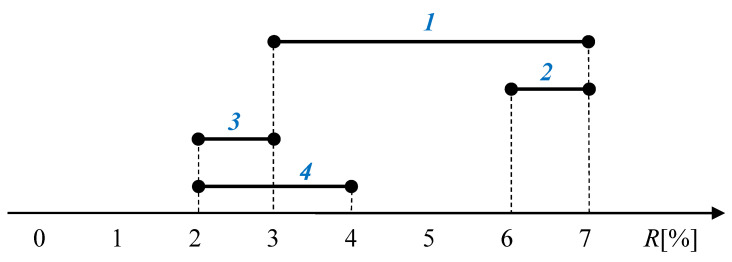
The returns of interval portfolios.

**Example** **1.** *Let us consider four interval-valued portfolio denoted as 1, 2, 3, and 4 with their predicted interval-valued returns $OPR_{1}$, $OPR_{2}$, $OPR_{3}$, and $OPR_{4}$ presented in Figure 1. A question arises: what are the levels of risk concerned with these portfolios? Obviously, in the spirit of Markowitz’s approach [1,3], we should treat the width ($\bar{OPR}-\underline{OPR}$) of interval portfolio as the measure of its risk, as the width may be naturally treated as a variance. Based on such a reasoning, we can conclude that the portfolio 1 is four times more risky than portfolios 2 and 3 as (${\bar{OPR}}_{1}-{\underline{OPR}}_{1}=7-3=4\%$, ${\bar{OPR}}_{2}-{\underline{OPR}}_{2}=3-2=1\%$, ${\bar{OPR}}_{3}-{\underline{OPR}}_{3}=4-3=1\%$). It is clear that such a result seems to be justified when comparing portfolios 1 and 2, but from common sense there is no doubt that portfolio 3 is much more risky than portfolio 1. In reality, the risk to earn an unacceptable low return from 3 is considerably greater than that of portfolio 1, although formally portfolio 1 is burdened by greater uncertainty than portfolio 3. Therefore, based on the above consideration, we can conclude that the treating an uncertainty as the measure of risk does not always coincide with common sense. The same conclusion we have obtained from comparison of portfolios 1 and 2. In the Markowitz’s spirit, the portfolio 1 is more risky than the portfolio 4, whereas in any case the portfolio 1 provides the greater possible return than the portfolio 4 (${\bar{OPR}}_{1}-{\underline{OPR}}_{1}>{\bar{OPR}}_{4}-{\underline{OPR}}_{4}$, but ${\underline{OPR}}_{1}>{\underline{OPR}}_{4}$ and ${\bar{OPR}}_{1}>{\bar{OPR}}_{4}$).*

Let us consider the famous Sharpe Ratio which is completely based on the mean-variance methodology. In its simplest form it can be presented as follows. *Sharpe Ratio* = $\frac{R}{\sigma }$, where *R* is the portfolio return and σ is its variance. If σ is treated as the measure of risk, it is intuitively obvious that then the greater is the *Sharpe Ratio* the better is the portfolio. Let us consider two portfolios (which for the sake of simplicity are assumed to be evenly distributed): the first with $R_{1}$ = 4, $\sigma_{1}$ = 2 and the second with $R_{2}$ = 1, $\sigma_{1}$ = 0.25. Then, we have $\frac{R_{1}}{\sigma_{1}}$ = 2 and $\frac{R_{2}}{\sigma_{2}}$ = 4 and we should recognize that the first portfolio is two times worse than the second one. Obviously this is an absurd result as the first portfolio is evenly distributed in the interval [2,6] whereas the second one is distributed in the interval [0.75,1.25]. It is clear that any reasonable person will choose the first portfolio. This contradiction may be explained by the proposition that a variance undoubtedly is the measure of something but not a measure of risk.

In addition, let us check the possibility of trade-off between the local criteria of portfolio return and risk presented by the variance. Obviously, a high risk (σ) may be re-compensated by a great return and a low return may be formally re-compensated by a low risk (low σ). On the other hand, a low risk (σ) means a high probability (certainty). Therefore, the last situation in terms of content may be described by the sentences “a certainly low return” or “a high probability of low return”. Therefore, in a meaningful sense we have no trade-off in such a case, and this is a consequence of treating the variance as a measure of risk. Therefore, taking also into account the critical opinions of other authors presented in the introduction, here we propose an approach to portfolio selection free of variance at all. In practice, investors don’t consider assets with a predicted failure.Therefore, only portfolios with positive or mostly positive future returns OPR are analyzing. In such a situation, only investor’s risk is the obtaining of unacceptable low return. Therefore, in the case of interval asset returns, the demand of such risk minimization may be formulated as $\underline{OPR}\to max$. On the other hand, the natural aspiration of investors is the earning as great as possible returns. From this point of view, the portfolio *4* (see Figure 1) seems to be more profitable than the portfolio *3* as it provides greater possible returns. That is why the demand to maximize possible returns may be formulated as $\bar{OPR}\to max$.

The above reasoning makes it possible to introduce formal mathematical definitions of main portfolio local criteria of risk minimization and return maximization.

It is easy to see from Equation (1) that the minimal value of $\underline{OPR}$ is ${\underline{OPR}}_{min}=\min\;\underline{ar_{i}}$ and the maximal value of $\bar{OPR}$ is ${\bar{OPR}}_{max}=\max\;\bar{ar_{i}}$ for *i* = 1 to *N*.

As $\underline{OPR}=\sum_{i=1}^{N}\underline{ar_{i}}as_{i}$, $\bar{OPR}=\sum_{i=1}^{N}\bar{ar_{i}}as_{i}$, we have always ${\underline{OPR}}_{min}\leq \underline{OPR}\leq \bar{OPR}\leq {\bar{OPR}}_{max}$. In the spirit of above analysis, the local criterion of portfolio’s risk may formulated as follows.

(2) $$PRisk=\frac{{\bar{OPR}}_{max}-\underline{OPR}}{{\bar{OPR}}_{max}-{\underline{OPR}}_{min}}$$

It is easy to see that the maximal value of PRisk equal to 1 we have when $\underline{OPR}={\underline{OPR}}_{min}$. Nevertheless, in the multiple criteria decision-making and optimization tasks it is more suitable to use instead of PRisk, the risk aversion 1−PRisk that decreases with lowering the ${\underline{OPR}}_{min}$. Therefore, for the risk aversion of portfolio (PARisk) we have

(3) $$PARisk=1-\frac{{\bar{OPR}}_{max}-\underline{OPR}}{{\bar{OPR}}_{max}-{\underline{OPR}}_{min}}.$$

Using the similar reasoning, the local criterion of portfolio profit maximization (OOPR) has been presented as follows.

(4) $$OOPR=1-\frac{{\bar{OPR}}_{max}-\bar{OPR}}{{\bar{OPR}}_{max}-{\underline{OPR}}_{min}}$$

We can say that the introduced criteria PARisk and OOPR reflect well our demands concerned with the behavior of local criteria of risk minimization and return maximization.

Let us consider the properties of introduced local criteria.

### 2.1. The Features of Proposed Criteria for the Valuation of Interval-Valued Portfolios

It is easy to prove that the values of *PARisk* and *OOPR* generally belong to the interval [0,1], and the critical values 0 and 1 can be obtained only in some hypothetical (asymptotic) cases which seem to be rather unrealistic ones. Nevertheless, to analyze the features of our approach, here we present some examples which make it possible to show that even in such asymptotic cases we obtain reasonable results.

Obviously, in practice we avoid negative returns. Nevertheless, if intervals *OPR* are not completely negative, but have negative parts, such situations cannot be excluded from our analysis.

Consider the illustrative examples.

All the results presented in Table 1 and Table 2 have reasonable explanations.

**Example** **2.**

**Table 1:** The values of *PARisk* and *OOPR* in the case of $\left[{\underline{OPR}}_{min},{\bar{OPR}}_{max}\right]$ = [1,5].

*OPR*	*PARisk*	*OOPR*
[1,4]	0	0.75
[1,5]	0	1
[2,4]	0.25	0.75
[2,5]	0.25	1

**Example** **3.**

**Table 2:** The values of *PARisk* and *OOPR* in the case of $\left[{\underline{OPR}}_{min},{\bar{OPR}}_{max}\right]$ = [−4,4].

*OPR*	*PARisk*	*OOPR*
[−4,4]	0	1
[−2,2]	0.25	0.75
[0,0]	0.5	0.5
[−1,2]	0.375	0.75
[−3,1]	0.126	0.625
[0,1]	0.5	0.625
[0,2]	0.5	0.75
[1,2]	0.625	0.75
[1,3]	0.625	0.875
[2,3]	0.75	0.875

We can see that *PARisk* is rising with rising of the left bound of interval *OPR* and *OOPR* is increasing with rising of the right bound of *OPR*.

This is in compliance with our propositions concerned with the formulation of local criteria of portfolio selection.

Using a few simple examples, let us look at the features of interval portfolios to make sure that they are logically consistent, reliable, and are in line with common sense.

**Example** **4.** *Let us consider that the three interval-valued portfolios $C_{1}$, $C_{2}$, and $C_{3}$ consist of four assets with the same sets of interval returns ($ar_{1}$,$ar_{2}$,$ar_{3}$,$ar_{4}$) and different sets of asset shares ($as_{1}$,$as_{2}$,$as_{3}$,$as_{4}$) (see Table 3). It is clear that $ar_{4}<ar_{1}<ar_{2}<ar_{3}$ (this can be strongly proved, e.g., using the method proposed in [57]).*

As a base of analysis of obtained results (see Table 3) we will use the values of *PARisk*, *OOPR* calculated for the portfolio $C_{1}$ with the uniform distribution of asset shares ($as_{i}=0.25$, *i* = 1 to *N*). In the portfolios $C_{2}$ and $C_{3}$, the asset shares 0.1, 0.2, 0.3, and 0.4 are used with different distributions.

In $C_{2}$, the greater asset shares are assigned to the greater asset returns: the maximal share (0.4) is assigned to the maximal asset return $ar_{3}=\left[5,10\right]$ and the minimal asset share (0.1) is assigned to the minimal asset return $ar_{4}=\left[0,2\right]$. It is easy to see that the portfolio $C_{2}$ is considerably better than portfolio $C_{1}$ as, considering the criteria of portfolio quality *PARisk*, *OOPR* are significantly greater in the case of $C_{2}$ than those in the case of $C_{1}$. An opposite situation we have in the case of portfolio $C_{3}$, where the greater asset shares are assigned to the assets with lower returns. In this case (portfolio $C_{3}$), the values of criteria *PARisk*, *OOPR* are substantially lower than those in the case of $C_{1}$.

Based on the above example, we can conclude that the well general portfolio formation policy in the case of interval asset returns should be the assigning greater shares to assets with greater interval returns.

Of course, this result is in line with common sense and logically justified.

Nevertheless, in Example 4 we have considered only the cases of intersection and lack of common area of interval asset returns. Therefore, in the next example, the situation when one interval return is completely included into another one is considered.

**Example** **5.** *To make our analysis more transparent, let us consider three portfolios D, E, and F (see Table 4) with only two assets such that one of them is completely included into another one ($ar_{5}\subset ar_{6}$). Then, using the method proposed in [57] we get $ar_{5}<ar_{6}$.*

As a base of comparison we use the portfolio *D*, in which equal shares are assigned to different assets, i.e., $as_{5}=as_{6}=0.5$. In the portfolios *E* and *F*, we have the opposite distributions of asset shares ($as_{5}=0.2$, $as_{6}=0.8$ for the portfolio *E* and $as_{5}=0.8$, $as_{6}=0.2$ for the portfolio *F*). In the portfolio *E*, the greater share is assigned to the greater asset ($as_{6}=0.8$). As a result, the decreasing of *PARisk* and increasing of *OOPR* in comparison with the values of these parameters in the portfolio *D* is observed in Table 4. The opposite situation is observed in the case of portfolio *F*, where $as_{5}=0.8$ (the increasing of *PARisk* and decreasing of *OOPR* in comparison with the results of the portfolio *D*).

The obtained results allow us to say that interval valued portfolio selection is a multiple criteria task and its optimal solution should be based on the compromise between competing local criteria.

At first glance, the results we obtained in this example make it possible to consider the results obtained for the portfolio with uniform distribution of asset shares as somewhat averaged (neutral) ones, but as it is shown in the next example this not always the case.

**Example** **6.** *In this example, we consider that the three portfolios (G, H, and K) consist of four assets with the equal sets of interval asset returns $ar_{7},ar_{8},ar_{9},ar_{10}$ and the different distributions of asset shares (see Table 5). Comparing the interval asset returns, we can find the cases of their inclusion $ar_{7}\subset ar_{8}$, $ar_{9}\subset ar_{10}$, intersection $ar_{8}⋂ar_{10}\neq \emptyset$ and the lack of common area (e.g., $ar_{7}⋂ar_{9})=\emptyset$). In other words, we can say that all possible in practice cases of mutual placement of interval returns are presented in the considered example.*

Similar to the previous examples, we choose the portfolio *G* with the uniform distribution of asset shares $as_{7}=as_{8}=as_{9}=as_{10}=0.25$ as the base of comparison. As $ar_{7}\subset ar_{8}$, $ar_{9}\subset ar_{10}$ and $ar_{8}>ar_{10}$, while $ar_{8}⋂ar_{10}=\emptyset$, the greatest shares (0.3 and 0.4) in portfolios *H* and *K* are assigned to the assets with the greatest returns ($ar_{7}$ and $ar_{8}$) and the smallest shares (0.1 and 0.2) are assigned to the assets with minimal returns ($ar_{9}$ and $ar_{10}$).

As in the previous example, we can see that rising of one criterion (e.g., *PARisk*) is accompanied by the decreasing of another one (*OOPR*). Nevertheless, in both analyzed cases (portfolios *H* and *K*) the greater values of both local criteria *PARisk* and *OOPR* than those we have got in the case of uniform asset shares distribution (see portfolio *G*) were obtained. This means that it is possible to find an optimal set of shares using an appropriate method for the solution of multiple criteria tasks.

### 2.2. The Bicriteria Interval Valued Portfolio Optimization

Let us define the vector of variables of considered optimization task as the vector of asset shares $\vec{as}=\left(as_{i}\right)$, *i* = 1 to *N*. Generally, the weights (the relative importance) $w_{PARisk}$ and $w_{OOPR}$ of our local criteria *PARisk*, *OOPR* can be included in the set of veriables. Nevertheless, we prefer here to consider these weights as the fixed parameters specified by an investor based on his/her own preferences concerned with aspirations of overall portfolio return maximization *OOPR*⇒max and risk minimization which in our case may be presented as the risk aversion maximization *PARisk*⇒max. Of course, it should be always $w_{PARisk}$ + $w_{OOPR}$ = 1. It was shown above that our local criteria *PARisk* and *OOPR* lay in the interval [0,1] and should be maximized.

In this paper, we will treat the portfolio selection as a multiple criteria optimization task in the form sometimes refereed to as scalarelized one [58]. This is an alternative approach to the portfolio optimization problem [59]. This approach aggregates local criteria (such as risk and profit criteria) ($c{\left(\vec{x}\right)}_{1}$, $c{\left(\vec{x}\right)}_{2}$,…,$c{\left(\vec{x}\right)}_{n}$), where $\vec{x}$ is a vector of decision variables, into one general criterion by assigning a weighting coefficient $w_{i}$ to each criterion. Then, a solution technique is to maximize a positively weighted convex sum of the local criteria, that is, Maximize $D\left(\vec{x}\right)=\sum_{i=1}^{n}w_{i}c{\left(\vec{x}\right)}_{i}$.

The concept of optimality in the multiple criteria optimization is equivalent to Pareto optimality. Therefore, a vector ${\vec{x}}^{o}$ is said to be Pareto optimal if and only if there is no $\vec{x}$ such that $c{\left(\vec{x}\right)}_{i}\leq c{\left({\vec{x}}^{o}\right)}_{i}$, *i* = 1, 2, …, n. It was proved [60] that ${\vec{x}}^{o}$ is the Pareto point if $D(\vec{x})$ achieves its maximal value. Therefore, varying the weights of local criteria, all Pareto optimal solutions may be obtained.

As the weights of local criteria may reflect their relative importance assigned by an investor, we can say that described approach seems to be more preferable than the classical one. Such a multiple criteria approach was successfully used for portfolio optimization in [61]. It is very important that the validity of the proposed approach was verified through an empirical testing application on the top 75 companies of Tehran Stock Exchange Market in 2017.

At first glance, the use of weighted sum of local criteria solves the problem. Nevertheless, there are important problems concerned with the use of weighted sum and some other popular methods for local criteria aggregation which were revealed in the fuzzy setting [62].

Somewhat ahead of events let us assume that we deal with two local criteria *A* and *B* dependent on *x* and represented by the symmetrical triangular fuzzy numbers with correspondent membership functions $\mu_{A}\left(x\right)$ and $\mu_{B}\left(x\right)$. It is clear that if they are not intersecting then we probably have two different single-criterion problems. Then, suppose we have *A* = (2,6,10) and *B* = (8,12,16). The Pareto region is the interval [8.10], where the decreasing of $\mu_{A}\left(x\right)$ is accompanied by the increasing of $\mu_{B}\left(x\right)$. Suppose that the criteria *A* = (2,6,10) and *B* = (8,12,16) are of equal importance. Then, the only one reasonable demand for $x^{o}$ to be the optimal solution is that in such a point the values of local criteria are equal once subject to they are as great as possible. Obviously $x^{o}=arg\left(maxmin\left(\mu_{A}\left(x\right),\mu_{B}\left(x\right)\right)\right)$, i.e., in our case $x^{o}=9$. On the other hand, using the weighted sum in the Pareto region we get $D_{ws}\left(x\right)=\frac{1}{2}\mu_{A}\left(x\right)+\frac{1}{2}\mu_{B}\left(x\right)=const$, i.e., the general criterion $D_{ws}\left(x\right)$ does not provide any Pareto optimal solution, whereas the min-criterion $D_{min}\left(x\right)=min\left(\mu_{A}\left(x\right),\mu_{A}\left(x\right)\right)$ and multiplicative criterion $D_{mul}\left(x\right)=\left(\mu_{A}\left(x\right)\mu_{B}\left(x\right)\right)$ provide the optimum $x^{o}=9$. It is shown in [62] that in the case of more complicated shapes of the triangular *A* and *B*, both the $D_{ws}\left(x\right)$ and $D_{mul}\left(x\right)$ general criteria provide the optimal solution strictly on the border of Pareto region. Of course, such a solution formally belongs to the Pareto optimal solutions, but in such an optimal point the values of $\mu_{A}\left(x\right)$ and $\mu_{B}\left(x\right)$ are different that is in contradiction with the assumption of their equal importance. In [62], it is shown that introducing different weights $w_{A}$ and $w_{B}$ does not improve the situation; moreover, the general criterion $D_{z}\left(x\right)=min\left(w_{A}\mu_{A}\left(x\right),w_{B}\mu_{B}\left(x\right)\right)$ proposed by Zimmerman [63] in this case provides the results that are not in line with common sense. However, there are no problems with the Yager’s min-type aggregation [64] (see below). It is worth noting that in some applications, e.g., in ecological modeling, the weighted sum aggregation is expressly forbidden to use [65] as there are important cases in practice when too low values of one local criterion cannot be recompensated by great values of other criteria.

Based on the above analysis, we can conclude that weighted sum aggregation is rather an unreliable method, but taking into account that it provides Pareto optimal solutions (as well as the other considered methods) and is very important for theoretical studies, e.g., being the cornerstone of the Utility theory, in the following we will use it in the analysis together with the other approaches. Taking into account that all aggregation methods have own advantages and limitations and that the choice of method for aggregation of local criteria is an application dependent problem [66], we will continue the formulation of our bicriteria optimization problem as follows.

To formulate the optimization task, the local criteria with their weights should be aggregated in a general criterion to be maximized to get an optimal vector of variables (asset shares).

In the literature [62,67], we can find many different approaches to the formulation of general criterion in multiple criterion optimization tasks. Among them, the most popular and frequently used in practice are the Yager’s ($D_{1}$), multiplication ($D_{2}$), and addition (weighted sum) ($D_{3}$) types of aggregation, which in our case may be presented as follows,

(5) $$D_{1}\left(\vec{as}\right)=minOOPR{\left(\vec{as}\right)}^{w_{OOPR}},\;PARisk{\left(\vec{as}\right)}^{w_{PARisk}},$$

(6) $$D_{2}\left(\vec{as}\right)=OOPR{\left(\vec{as}\right)}^{w_{OOPR}}PARisk{\left(\vec{as}\right)}^{w_{PARisk}},$$

(7) $$D_{3}\left(\vec{as}\right)=w_{OOPR}OOPR\left(\vec{as}\right)\;+\;w_{PARisk}PARisk\left(\vec{as}\right).$$

The presented methods of aggregation (5–7) may be used solely and in different combinations. Their advantages and limitations were studied in [62], where it is shown that the most reliable approach is the Yager’s type of aggregation ($D_{1}$) [64], the multiplicative aggregation ($D_{2}$) seems to be less reliable and the most popular additive (weighted sum) aggregation ($D_{3}$) may provide wrong counterintuitive results in the Pareto area. Nevertheless, all method of aggregation have own advantages and drawbacks and it is often impossible to choose the best one for the solution of real-world optimization problem. That is why, if we have a complicated multiple-objective problem, all relevant methods for aggregation of local criteria should be used. If the results of the solution of optimization task we got using different aggregation modes are comparable we can say that they can be treated as optimal ones. In the opposite case, the compromise solution may be obtained using the method of aggregation of aggregating modes based on the synthesis of type 2 and level 2 fuzzy sets proposed in [67], but this is out of scope of the current paper. Therefore, in our case the optimization problem may be formulated as follows,

(8) $${\vec{as}}_{opt}=arg\left(maxD_{k}\left(\vec{as}\right)\right)\;\left(k=1,2,3\right)\;s.t.,\;\sum_{i=1}^{N}as_{i}=1.$$

For the solution of formulated problem, the modification of the direct random search method [68] taking into account the restrictions $\sum_{i=1}^{N}as_{i}=1$ and $0\leq as_{min}\leq as_{i}\leq as_{max}\leq 1$, where the values of $as_{min}$ and $as_{max}$ are defined by an investor was used.

Of course, there are many other modern optimization methods, e.g., genetic algorithms developed in last decades. Nevertheless, based of a number of persuasive examples, it was shown in [69] that when we are dealing with the complex nonlinear optimization problem, the direct search methods perform significantly better.

**Example** **7.** *In this example, we will use the portfolios based on the four assets with returns which are the same as in Example 6. Varying the values of local criteria weights ($w_{PARisk}$ and $w_{OOPR}$) on three levels we have obtained for three aggregation methods ($D_{1}-D_{3}$) the nine optimization tasks, the solutions of which are presented in Table 6 in the form of optimal portfolios $L_{1}$−$L_{9}$.*

*In the optimization, the following restrictions were applied, $as_{min}$ = 0.01, $as_{max}$ = 0.97, i.e., $0.01\leq as_{i}\leq 0.97$. The non-optimal values of general criteria ($D_{i}$, i = 1 to 3) for the portfolios with asset returns and shares from the portfolios G, H, and K (see Example 6) are presented in Table 7 for the local criteria weights which are the same as in Table 6. In Table 6, $D_{i}\left(as_{opt}^{\to }\right)$ are the values of general criteria $D_{i}$ in the points of optimal solution ($as_{opt}^{\to }$).*

We can see that the optimal solutions $as_{opt}^{\to }$ depend on the values of local criteria weights ($w_{PARisk}$ and $w_{OOPR}$) and the used method of local criteria aggregation (see Table 6, portfolio $L_{1}-L_{9}$). The optimal portfolios are strongly dominated by the assets with the maximal interval returns ($ar_{7}$ and $ar_{8}$), whereas the shares of assets with minimal interval returns ($ar_{9}$ and $ar_{10}$) are equal to the accepted permissible minimal value, $as_{min}$ = 0.01. This result seems to be quite natural as the intervals $ar_{7}$ and $ar_{8}$ are significantly greater than the intervals $ar_{9}$ and $ar_{10}$. Besides, as the portfolios $L_{1}-L_{9}$ are optimal ones they are characterized by the substantially greater values of general criteria ($D_{i}\left(\vec{as}\right)$) than those obtained from portfolios *G*, *H*, and *K* (see Table 7). Let us denote the value of general criterion for *i*th type of aggregation and *P*th portfolio as $D_{i}\left(P\right)$. Then, using the methods of interval comparison presented in [57], from Table 6 and Table 7 we have obtained $D_{1}\left(L_{3}\right)=0.80>D_{1}\left(K\right)=0.70$ (max{$D_{1}\left(L_{1}\right)$, $D_{1}\left(L_{2}\right)$, $D_{1}\left(L_{3}\right)\}$>max{$D_{1}\left(G\right)$, $D_{1}\left(H\right)$, $D_{1}\left(K\right)\}$), $D_{2}\left(L_{6}\right)=0.69>D_{2}\left(H\right)=0.54$ (max{$D_{2}\left(L_{4}\right)$, $D_{2}\left(L_{5}\right)$, $D_{2}\left(L_{6}\right)\}$>max{$D_{2}\left(G\right)$, $D_{2}\left(H\right)$, $D_{2}\left(K\right)\})$ and $D_{3}\left(L_{9}\right)=0.78>D_{3}\left(H\right)=0.58$ (max{$D_{3}\left(L_{7}\right)$, $D_{3}\left(L_{8}\right)$, $D_{3}\left(L_{9}\right)\}$>max{$D_{3}\left(G\right)$, $D_{3}\left(H\right)$, $D_{3}\left(K\right)\}$).

These results indicate the effectiveness of the proposed method for optimizing the interval portfolio.

It is seen that the optimal portfolios $L_{1}-L_{9}$ are characterized by the low diversification. This is caused by the use of extremely wide range of permissible values of shares $as_{min}$ = 0 and $as_{max}$ = 1 in the optimization procedure.

**Example** **8.** *To extend the diversification of optimized portfolios, in this example we used the following range of admissible values of shares ($0.05\leq as_{i}\leq 0.40$). As in the previous example, the four assets with returns the same as in Example 6 were used. Similar to the previous example, the nine optimal portfolios $M_{1}$–$M_{9}$ were obtained. The results are presented in Table 8.*

Based on the reasoning as in the previous example, the domination of the assets with maximal interval returns $ar_{7}$ and $ar_{8}$ in all optimal portfolios $M_{1}$–$M_{9}$ (see Table 8) can be easily explained. The order of positions held by assets in terms of their share in the portfolio depends on the type of aggregation of local criteria used and their weights. In the portfolios $M_{1}$–$M_{5}$ and $M_{8}$, we have $as_{7}>as_{8}>as_{9}>as_{10}$, whereas in the portfolios $M_{6}$, $M_{7}$, and $M_{9}$ we observe $as_{8}>as_{7}>as_{10}>as_{9}$. These results can be explained by analyzing the values of asset returns taking into account the weights of local criteria and the values of general criteria. This can best be illustrated by analyzing the optimal shares obtained using the weighted sum aggregation ($D_{3}$) for the assets with maximal returns $ar_{7}$ and $ar_{8}$. We can see that in the portfolios $M_{7}$ and $M_{9}$ we have $as_{8}>as_{7}$. This result is caused by using the weights $w_{OOPR}$ (0.5 and 0.7, respectively); however, first of all by the large value of the right border of the interval $ar_{8}$ such that ${\bar{ar}}_{8}>{\bar{ar}}_{7}$. An opposite situation we have in the portfolio $M_{8}$, in which the used large weight $w_{PARisk}$ = 0.9 together with the fact that ${\underline{ar}}_{7}>{\underline{ar}}_{8}$ predetermine the domination of asset with $as_{7}$.

As it should be, the lowering of $as_{max}$ from 1 to 0.4 caused the decreasing the valued of optimized general criteria $D_{i}\left(as_{opt}^{\to }\right)$ in comparison with those in the previous example. We can see that $D_{1}\left(M_{3}\right)=0.72<D_{1}\left(L_{3}\right)=0.8$, $D_{2}\left(M_{6}\right)=0.58<D_{2}\left(L_{6}\right)=0.69$, $D_{3}\left(M_{9}\right)=0.62<D_{3}\left(L_{9}\right)=0.78$ (see Table 7 and Table 8).

## 3. The Bicriteria Optimization of Fuzzy Portfolio

In this section, we propose an approach to the fuzzy portfolio optimization based on the α-cut presentation of fuzzy values and the fuzzy extension of method for the interval-valued portfolio selection described in the previous section.

In the considered case of fuzzy portfolio optimization, we first deal with fuzzy asset returns $ar_{Fi}$, *i* = 1 to *N*. They can be presented by sets of α-cuts, which are defined as follows.

Let *X* be a fuzzy value, then it can be presented as $X=\underset{\alpha }{⋃}\alpha X_{\alpha }$, where $\alpha X_{\alpha }$ is the fuzzy subset x∈U, $\mu_{X}\left(x\right)\geq \alpha$, $X_{\alpha }$ is the support set of fuzzy subset $\alpha X_{\alpha }$, and *U* is the universe of discourse. It is important that $X_{\alpha }$ is a crisp interval.

Therefore, for the α-cut presentation of the fuzzy asset returns we have $ar_{Fi}=\underset{\alpha }{⋃}\alpha ar_{Fi\alpha }$, where $ar_{Fi\alpha }$ are intervals $\left[\underline{ar_{Fi\alpha }},\bar{ar_{Fi\alpha }}\right]$.

For the sake of simplicity, here we will consider the widely used in practice trapezoidal fuzzy values which can be represented by two intervals on the support and the core of trapezes, i.e., by the quadruples $\left[\underline{ar_{Fi0}},\underline{ar_{Fi1}},\bar{ar_{Fi1}},\bar{ar_{Fi0}}\right]$.

The expressions (3) and (4) for calculation of local criteria may be presented in the fuzzy case by α-cuts as follows,

(9) $$PARisk_{\alpha }=1-\frac{{\bar{OPR}}_{\alpha \;max}-{\underline{OPR}}_{\alpha }}{{\bar{OPR}}_{\alpha \;max}-{\underline{OPR}}_{\alpha \;min}},$$

(10) $$OOPR_{\alpha }=1-\frac{{\bar{OPR}}_{\alpha \;max}-{\bar{OPR}}_{\alpha }}{{\bar{OPR}}_{\alpha \;max}-{\underline{OPR}}_{\alpha \;min}},$$

where ${\underline{OPR}}_{\alpha \;min}=\min\;\underline{ar_{Fi\alpha }}$, ${\bar{OPR}}_{\alpha \;max}=\max\;\bar{ar_{Fi\alpha }}$,

$\underline{OPR_{\alpha }}=\sum_{i=1}^{N}\underline{ar_{Fi\alpha }}as_{i}$, ${\bar{OPR}}_{\alpha }=\sum_{i=1}^{N}\bar{ar_{Fi\alpha }}as_{i}$.

Finally, the values of local criteria are calculated as the weighted sums on α-cuts assuming that the contribution of α-cut to the overall estimation is rising (in the simplest linear way) with increasing the value of α:

(11) $$PARisk=\frac{\sum_{\alpha }PARisk_{\alpha }\alpha }{\sum_{\alpha }\alpha },\;OOPR=\frac{\sum_{\alpha }OOPR_{\alpha }\alpha }{\sum_{\alpha }\alpha }$$

Then, the optimal asset shares $as_{opt}^{\to }$ for the three considered methods for local criteria aggregation are obtained from (5)–(8) with $PARisk(\vec{as})$ and $OOPR(\vec{as})$ from (11).

**Example** **9.** *In this example, we consider the optimized fuzzy portfolios $P_{1}$–$P_{6}$ (see Table 9) presented by the fuzzy asset returns $ar_{F7}$, $ar_{F8}$, $ar_{F9}$, and $ar_{F10}$, with the supports the same as the interval-valued returns $ar_{7}$, $ar_{8}$, $ar_{9}$, and $ar_{10}$ from the previous example. The results of optimization are presented in Table 9.*

*We can see that the obtained optimal asset shares do not depend on the methods for the local criteria aggregation and their weights. Only the values of $as_{min}$ and $as_{min}$ effect the results of optimization (the observed different values of general criteria calculated for the same optimal asset shares $\vec{as}$ are the consequence of using the different local criteria weights in the expressions (5)–(7)). These results can be easily explained by the choice of the supports of fuzzy returns, which provide the strong domination of the assets with $ar_{F7}$, $ar_{F8}$ over those with $ar_{F9}$ and $ar_{F10}$. No wonder the obtained results are practically the same as in the previous example with interval asset returns $ar_{7}$, $ar_{8}$, $ar_{9}$ and $ar_{10}$. This indirectly confirms the validity of the proposed method.*

Above we have assumed that the weights (importance) of local criteria $w_{PARisk}$ and $w_{OOPR}$ may be assigned by an expert based on his/her own preferences and experience, but this is not always the case. Therefore, in situations when there are no such predetermined values of weights, they may be included in the set of decision variables. As the result, a new optimization task, which may be called a “full’ optimization, is formulated. The results of its solution in the considered example are presented in Table 10. We can see that the aggregations $D_{1}$–$D_{3}$ provide identical results of optimization, but $D_{2}$ and $D_{3}$ delivered these results with optimal values of $w_{PARisk}$ and $w_{OOPR}$, which are substantially different from those obtained using $D_{1}$. This is in qualitative compliance with the theoretical analysis [62] and our informal reasoning at the beginning of Section 2.2. The asset shares obtained based on “full” optimization do not differ from those presented in Table 9.

**Example** **10.** *In the above examples of interval and fuzzy portfolio optimization, we considered the portfolios consist of four assets the two of them had significantly (even overwhelmingly) greater returns then those of the two remaining assets. Of course, in such extreme and simple situations we have obtained that the optimal portfolios consist practically of two the most profitable assets and the obtained results occurred to be nearly independent on the methods for local criteria aggregation and their weights.*

*Meanwhile, in practice, investors always intent to select for their portfolios the assets with at least commensurate returns.*

**Figure 2:**
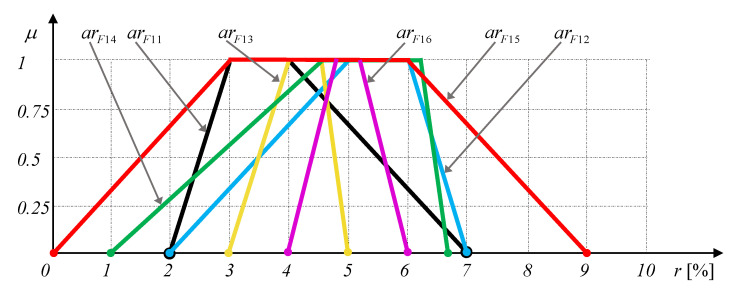
Trapezoidal fuzzy asset returns (Example 10).

*Therefore, in this example, we will consider optimized portfolios based on the set of comparable trapezoidal fuzzy asset returns presented in Figure 2 and Table 11.*

The six sets of optimal portfolios $U_{1}$–$U_{6}$, each of which in turn consist of three optimal portfolios $\vec{as}=\left\{as_{11},as_{12},as_{13},as_{14},as_{15},as_{16}\right\}$ obtained for the three types of local criteria aggregation (5)–(7) for different values of local criteria weights, $as_{min}$ and $as_{max}$ are presented in Table 11.

Opposite to the Example 9, we can see an indisputable dependence of optimal solutions on the method for local criteria aggregation, local criteria weights, $as_{min}$, and $as_{max}$. Moreover, in some cases (see, e.g., the set $U_{1}$) the qualitative contradictions between optimal solutions obtained for different types of local criteria aggregation and the same other parameters take place. Of course, in such cases the use of above-mentioned method for aggregation of aggregating modes [67] may be a good solution of problem. We can see that the assets with $ar_{12}$ and $ar_{16}$ dominate over other ones as they have considerable greater returns (in a fuzzy sense [57]) than the other assets in the portfolio. We can see that these dominating assets are also the competing ones as the asset with $as_{16}$ is only slightly greater than that with $ar_{12}$. Summarizing, we can say that all the results presented in Table 11 are in line with common sense and can be explained based mainly by the methods for comparison of fuzzy values.

As in the previous example, here we provide also the results of “full” optimization obtained by inclusions of local criteria weights in the set of decision variables, see Table 12.

When comparing the results presented in Table 11 and Table 12, we can see that the use “full” optimization makes it possible to avoid the problem of high dependence of results on the applied method for aggregation as in Table 11. Besides, opposite to the Example 9, the difference between the optimal criteria weights $w_{PARisk}$ and $w_{OOPR}$ obtained using $D_{2}$, $D_{3}$ and $D_{1}$ is rather ineligible.

**Example** **11.** *In this example, we compare the results obtained using our method with those from [70] where the fuzzy portfolios consist of five assets $C_{1}$–$C_{5}$ were considered. The returns of these assets were assumed in [70] to be normal fuzzy values with membership functions $\mu \left(r\right)=exp\left(-\frac{{\left(r-m\right)}^{2}}{w^{2}}\right)$, where m is the mean (center) and w is the spread of distribution, the values of which are presented in Table 13.*

The seven widely reputed methods (their names are presented in Table 14) were applied in [70] to the solution of fuzzy portfolio optimization problems with the parameters shown in Table 13.

Here, we provide a short description of these methods.

The original model of the portfolio selection problem was proposed by Markowitz [3]. The model is the so-called *V*-model [71] in stochastic programming. To obtain a Pareto optimal solution, he treated the problem so as to minimize the variance keeping the expected value at a given constant τ, i.e., respect to τ = 0.18 indicates a 17 percent investment in the fifth asset which may be regarded as inferior (see Table 14).

Kataoka’s model [72,73] is based on random return rates. In this model, we maximize *z* such that the probability of the event that the total return rate is not smaller than *z* is at least 1 −α, where α corresponds to α-fractile of the standard normal distribution. The result presented in Table 14 was obtained applying this model with α = 0.05.

The next model is the minimum-risk model with random return rates [71]. In contrast to Kataoka’s model, the minimum-risk model maximizes the probability of the event that the total return rate is not smaller than a predetermined value $z_{0}$ in this model. In Table 14, the result obtained with $z_{0}$ = 0.18 is presented.

The Spread minimization fuzzy model was formulated in [70]. It is based on the minimization of weighted sum of assets shares, where the weights are spreads of the normal fuzzy numbers presenting the fuzzy assets returns subject to the portfolio return is not smaller than the model parameter τ. In Table 14, the result obtained with τ = 0.18 is shown.

The fractile approach corresponds to Kataoka’s model [71,72] of a stochastic programming problem. This approach was formulated in [70] as a possibilistic model. The model is based on the maximization of parameter *z* subject to a Necessity degree (Nes) to what extent a portfolio return is not smaller than *z* is greater than $h_{0}$, where $h_{0}$ is a predetermined value in the interval [0,1]. The result obtained using this model with $h_{0}$ = 0.9 is presented in Table 14.

The modality optimization model corresponds to the minimum-risk approach to a stochastic programming problem [71]. In [70], it was presented in the possibility setting as the maximization of Necessity degree (Nes) to what extent a portfolio return is greater than $z_{0}$ where $z_{0}$ is in the interval [0,1]. The result obtained with $z_{0}$ = 0.18 is shown in Table 14.

The Minimax regret model is the most complicated possibilistic model among those considered in this paper and therefore needs too many mathematic expressions and explanations to be presented in the current paper, which is not devoted to the possibilistic portfolio selection. It is worth noting here that this model is based on the maximization of the so-called Regret criterion, which is the difference between the optimal total return rate and the obtained total return rate. This criterion is a possibilistic variable represented by a corresponding possibility distribution. More details may be found in [70,74].

The details concerned with the strong definitions of used methods in the fuzzy and possibilistic settings may be found in [70]. It is important to note also that all solutions ($as_{1}$–$as_{5}$) presented in Table 14 are Pareto optimal [70]. We can see that competing methods provide a complete set of possible portfolios diversification. The result provided by Markowitz’s model seems to be a dubious one, as it is impossible to justify in the reasonable way the dominance of $C_{4}$ over all other assets. At the same time, the most profitable assets $C_{1}$–$C_{3}$ present simultaneously in four of seven portfolios and the pair of even more profitable assets $C_{1}$–$C_{2}$ presents in the five portfolios.

Therefore, when analyzing the results from Table 14 as a whole, we can conclude that the most reliable portfolio should consist of only two portfolios $C_{1}$ and $C_{2}$.

We can say that we have made the same conclusion based on the results obtained using our bicriteria method. At the end, it is worth noting that methods analyzed in [70] are by their nature the single-criterion ones, being based on the maximization or minimization of one local criterion (return or risk), whereas the other one serves only as a restriction. Of course, this considerably limits the practical applicability of these methods.

When using our bicriteria method for optimization of portfolio which consist of five assets $C_{1}-C_{5}$, their normal fuzzy returns with parameters presented in Table 13 were approximated by sets of corresponding α-cuts. In the optimization procedure, the extremal values of shares $as_{min}$ = 0 and $as_{max}$ = 1 were used as they provide the natural limitations on permissible solutions.

The obtained results are presented in Table 15, Table 16 and Table 17. In Table 17, we can see that the weighted sum aggregation of local criteria ($D_{3}$) provides rather trivial results (which however properly reflect changes of local criteria weights). This is in compliance with our theoretical results (see [62]). Therefore, we do not recommend to use this type of aggregation in the portfolio selection.

Obtained results show that in our case optimal portfolios are consist of only two assets $C_{1}$ and $C_{2}$ independently on the used method for local criteria aggregation and their weights. Of course, decreasing the value of $as_{max}$ = 1 we can obtain even completely diversified portfolios consist of all $C_{1}-C_{5}$, but they will be considerably less profitable than obtained two-assets portfolios (see also Examples 9 and 10 for more detail). Of course, the features of two-assets portfolios are more easy to analyze, but we will show that in our case namely such portfolios reflect better the preferences of real cautious investor based on his/her attitude to the compromise between future profits and risks.

From Table 15 and Table 16, and in some sense from Table 17, we can see that the optimal values of share $as_{2}$ are rising in line with lowering the share $as_{1}$ when the value of risk minimization local criterion weight is rising in relation to the lowering of profit maximization local criterion weight. As, based on analysis of Table 13, we can conclude that $C_{2}$ is less profitable and considerably less risky than $C_{1}$, it seems quite natural that the share of less risky asset should rise in line with increasing the weight of the risk minimization criterion.

We can see that opposite to the great difference of used local criterion weights, obtained fuzzy portfolios are mostly similar ones. Nevertheless, the share of more profitable asset ($as_{1}$) and the mean value of optimal fuzzy portfolio return are lowering along with decreasing the weight of profit maximization criterion.

It was shown above (see Examples 9 and 10) that the proposed methods, from those based on the simple view to those based on the fuzzy portfolio selection problem, may provide widely diversified portfolios. Nevertheless, in considering the examples, the wide diversification seems to be undesirable as it does not reflect the specificity of the real investor’s reasoning. This is easy to explain. Based on the simple reasoning, the asset $C_{3}$ cannot be included in the portfolio. The assets $C_{3}$ and $C_{2}$ have the same spreads and therefore may be treated as equally risky ones. On the other hand, the mean value of fuzzy return of $C_{2}$ is greater than that of $C_{3}$. Then, as we deal with the normal symmetrical distributions we can say that in any case the asset $C_{2}$ should be preferred. Then, it seems to be obvious that in a presence of the asset $C_{2}$ the only natural policy of investor is the rejection of the asset $C_{3}$ from the consideration. It is important that the most of optimal portfolios obtained using the known models (see Table 14) include the asset $C_{3}$. The only exception is the Spread min model that provides the portfolio consist of assets $C_{1}$ and $C_{2}$, but opposite to our method, this model does not take into account the investor’s real preferences concerned with local criteria relative importance (weights). Further, the mean value of asset $C_{1}$ return is 40 % greater than that of asset $C_{4}$ and 5 times greater than that of asset $C_{5}$. In this way, a cautious investor investor should reject from the use the assets $C_{4}$ and $C_{5}$ in the portfolio.

Therefore, we can say that the features of our method are logically justified and in line with common sense. The proposed method based on a simple view on the interval and fuzzy portfolio selection problem have tangible advantages in comparison with known methods.

Then, we can say that results presented in Table 15, Table 16 and Table 17 that were obtained based on the proposed new concept of the local criteria of interval and fuzzy portfolio optimization in the synthesis with the bicriteria approach, better reflect the nature of portfolio optimization than those obtained using known methods for the fuzzy portfolio selection.

As in two previous examples, in Table 18, we present the result of “full” optimization, i.e., obtained by including the local criteria weights in the set of decision variables.

It is seen that in the considered example, the optimal asset shares practically do not depend on the choice of aggregating method, but significantly depend in the diversification level. The obtained results reflect the specificity of considered task (see Table 17) manifested in the strong dominance of profit criterion over risk one. It is important that such a domination is hard to be revealed in the presiding analysis before the solution of optimization task.

In this paper, we have compered the developed method only with the simple known methods which can be treated (in some sense) as asymptotic ones, because only the such type of study allows us to reveal and present transparently the specificity of a new method. The use of more complicated methods as a base of comparison, e.g., as proposed in [23,34], does not provide such a possibility, as it is very hard to explain reasonable the inevitable difference between the results of competing methods. Only that we can say in the such situation is that this difference may considered as an evidence in favor of our method as more methodologically justified. It is worthy to note that in our approach the general criterion is formulated in the form of local criteria aggregation the number of which may be greater that two. Therefore, the proposed method may be easily extended by the inclusion additional criteria such as stock’s liquidity, transaction costs and so on.

## 4. Conclusions

In this paper, it is shown that the variance in the Markowitz’s mean-variance model for the portfolio selection with its numerous modifications does not always adequately represent the portfolio’s risk. Therefore, in the current paper, a new but simple view on the portfolio selection problem, which makes it possible to use a new approach to the formulation of local criteria of portfolio optimization is introduced. Based on this approach, an alternative treating of portfolio risk as the measure of possibility to earn unacceptable low returns of portfolio is used. A simple mathematical formalization of this measure is proposed. In a similar way, the criterion of portfolio’s return maximization as the measure of possibility to get a maximal return is introduced. Then, the portfolio optimization problem is formulated as a bicriteria optimization task and using critical examples, the features of the developed approach are studied. The α-cut representation of fuzzy returns is used.

To validate the proposed method, the results we got using it were compared with those obtained with the use of fuzzy versions of seven widely reputed methods for portfolio selection. As in the proposed approach we deal with the bicriteria task, the three most popular methods for local criteria aggregation are compared using the known example of fuzzy portfolio consist of five assets. Based on the results of provided analysis, it is established that the features of proposed method are logically justified and in line with common sense. The proposed method based on the simple view on the interval and fuzzy portfolio selection problem have tangible advantages in comparison with known methods.

It is shown that the results obtained using the proposed approach to the fuzzy portfolio selection reflect better the essence of this task than those obtained by widely reputed and popular traditional methods for the fuzzy portfolio selection.

As the generalized criterion is formulated as the convolution of local criteria, the method may be easily extended by inclusion of additional criteria such as stock’s liquidity, transaction costs, and so on.

## Figures and Tables

**Table 3:** Example 4.

${\mathrm{ar}}_{i}$	Portfolio $C_{1}$	Portfolio $C_{2}$	Portfolio $C_{3}$
[%]			
$ar_{1}=\left[2,5\right]$	$as_{1}=0.25$	$as_{1}=0.2$	$as_{1}=0.3$
$ar_{2}=\left[3,7\right]$	$as_{2}=0.25$	$as_{2}=0.3$	$as_{2}=0.2$
$ar_{3}=\left[5,10\right]$	$as_{3}=0.25$	$as_{3}=0.4$	$as_{3}=0.1$
$ar_{4}=\left[0,2\right]$	$as_{4}=0.25$	$as_{4}=0.1$	$as_{4}=0.4$
$\underline{OPR_{min}}$	0.0	0.0	0.0
$\underline{OPR}$	2.5	3.3	1.7
$\bar{OPR}$	6.0	7.3	4.7
$\bar{OPR_{max}}$	10.0	10.0	10.0
*PARisk*	0.25	0.33	0.17
*OOPR*	0.60	0.73	0.47

**Table 4:** Example 5.

${\mathrm{ar}}_{i}$	Portfolio D	Portfolio E	Portfolio F
[%]			
$ar_{5}=\left[3,5\right]$	$as_{5}=0.5$	$as_{5}=0.2$	$as_{5}=0.8$
$ar_{6}=\left[1,8\right]$	$as_{6}=0.5$	$as_{6}=0.8$	$as_{6}=0.2$
$\underline{OPR_{min}}$	1.0	1.0	1.0
$\underline{OPR}$	2.0	1.4	2.6
$\bar{OPR}$	6.5	7.4	5.6
$\bar{OPR_{max}}$	8.0	8.0	8.0
*PARisk*	0.14	0.06	0.23
*OOPR*	0.79	0.91	0.66

**Table 5:** Example 6.

${\mathrm{ar}}_{i}$	Portfolio G	Portfolio H	Portfolio K
[%]			
$ar_{7}=\left[5,7\right]$	$as_{7}=0.25$	$as_{7}=0.3$	$as_{7}=0.4$
$ar_{8}=\left[3,10\right]$	$as_{8}=0.25$	$as_{8}=0.4$	$as_{8}=0.3$
$ar_{9}=\left[1,2\right]$	$as_{9}=0.25$	$as_{9}=0.1$	$as_{9}=0.2$
$ar_{10}=\left[0,4\right]$	$as_{10}=0.25$	$as_{10}=0.2$	$as_{10}=0.1$
$\underline{OPR_{min}}$	0.0	0.0	0.0
$\underline{OPR}$	2.25	2.8	3.1
$\bar{OPR}$	5.75	7.1	6.6
$\bar{OPR_{max}}$	10.0	10.0	10.0
*PARisk*	0.225	0.28	0.31
*OOPR*	0.575	0.71	0.66

**Table 6:** Optimal solutions (Example 7).

i	$D_{i}$	$w_{\mathrm{PARisk}}$	$w_{\mathrm{OOPR}}$	$L_{i}$	$D_{i}\left(as_{opt}^{\to }\right)$	$as_{opt}^{\to }$			
						${\mathrm{as}}_{7\mathrm{opt}}$	${\mathrm{as}}_{8\mathrm{opt}}$	${\mathrm{as}}_{9\mathrm{opt}}$	${\mathrm{as}}_{10\mathrm{opt}}$
1	$D_{1}$	0.5	0.5	$L_{1}$	0.70	0.97	0.01	0.01	0.01
		0.9	0.1	$L_{2}$	0.53	0.97	0.01	0.01	0.01
		0.3	0.7	$L_{3}$	0.80	0.87	0.11	0.01	0.01
2	$D_{2}$	0.5	0.5	$L_{4}$	0.58	0.91	0.07	0.01	0.01
		0.9	0.1	$L_{5}$	0.51	0.97	0.01	0.01	0.01
		0.3	0.7	$L_{6}$	0.69	0.01	0.97	0.01	0.01
3	$D_{3}$	0.5	0.5	$L_{7}$	0.64	0.01	0.97	0.01	0.01
		0.9	0.1	$L_{8}$	0.51	0.97	0.01	0.01	0.01
		0.3	0.7	$L_{9}$	0.78	0.01	0.97	0.01	0.01

**Table 7:** Non-optimal values of general criterion for the portfolios from Example 6.

i	${\mathrm{ar}}_{i}$	$\vec{\mathrm{as}}$		Portfolio		
	[%]			G	H	K
7	$ar_{7}=\left[5,7\right]$	$as_{7}$		0.25	0.3	0.4
8	$ar_{8}=\left[3,10\right]$	$as_{8}$		0.25	0.4	0.3
9	$ar_{9}=\left[1,2\right]$	$as_{9}$		0.25	0.1	0.2
10	$ar_{10}=\left[0,4\right]$	$as_{10}$		0.25	0.2	0.1
i	Aggregation method	$w_{PARisk}$	$w_{OOPR}$	$D_{i}\left(\vec{as}\right)$		
1	$D_{1}$	0.5	0.5	0.47	0.53	0.56
		0.9	0.1	0.26	0.32	0.35
		0.3	0.7	0.64	0.68	**0.70**
2	$D_{2}$	0.5	0.5	0.36	0.45	0.45
		0.9	0.1	0.25	0.31	0.33
		0.3	0.7	0.43	**0.54**	0.53
3	$D_{3}$	0.5	0.5	0.40	0.50	0.49
		0.9	0.1	0.26	0.32	0.35
		0.3	0.7	0.47	**0.58**	0.56

**Table 8:** The results of interval valued portfolio optimization (Example 8).

Aggregation	$D_{i}\left(\vec{\mathrm{as}}\right)$	$w_{\mathrm{PARisk}}$	$w_{\mathrm{OOPR}}$	$\vec{\mathrm{as}}$				$M_{i}$
Method				${\mathrm{as}}_{7}$	${\mathrm{as}}_{8}$	${\mathrm{as}}_{9}$	${\mathrm{as}}_{10}$	
	0.58	0.5	0.5	0.40	0.39	0.16	0.05	$M_{1}$
$D_{1}$	0.37	0.9	0.1	0.40	0.39	0.16	0.05	$M_{2}$
	**0.72**	0.3	0.7	0.40	0.39	0.16	0.05	$M_{3}$
	0.49	0.5	0.5	0.40	0.39	0.16	0.05	$M_{4}$
$D_{2}$	0.36	0.9	0.1	0.40	0.39	0.16	0.05	$M_{5}$
	**0.58**	0.3	0.7	0.39	0.40	0.05	0.16	$M_{6}$
	0.53	0.5	0.5	0.39	0.40	0.05	0.16	$M_{7}$
$D_{3}$	0.37	0.9	0.1	0.40	0.39	0.16	0.05	$M_{8}$
	**0.62**	0.3	0.7	0.39	0.40	0.05	0.16	$M_{9}$

**Table 9:** The results of portfolio optimization (Example 9).

D	i	${\mathrm{ar}}_{\mathrm{Fi}}\left[\%\right]$	Portfolios					
			$P_{1}$	$P_{2}$	$P_{3}$	$P_{4}$	$P_{5}$	$P_{6}$
			${\mathrm{as}}_{i}$	${\mathrm{as}}_{i}$	${\mathrm{as}}_{i}$	${\mathrm{as}}_{i}$	${\mathrm{as}}_{i}$	${\mathrm{as}}_{i}$
$D_{1}$	7	[5,6,6.2,7]	0.94	0.40	0.94	0.40	0.89	0.40
	8	[3,3.2,3.4,10]	0.03	0.39	0.03	0.39	0.08	0.39
	9	[1,1.4,1.6,2]	0.01	0.05	0.01	0.05	0.01	0.05
	10	[0,3.6,3.8,4]	0.02	0.16	0.02	0.16	0.02	0.16
	$D_{1}\left(\vec{as}\right)$		0.79	0.63	0.66	0.44	0.86	0.76
$D_{2}$	7	[5,6,6.2,7]	0.94	0.40	0.94	0.40	0.94	0.40
	8	[3,3.2,3.4,10]	0.03	0.39	0.03	0.39	0.03	0.39
	9	[1,1.4,1.6,2]	0.01	0.05	0.01	0.05	0.01	0.05
	10	[0,3.6,3.8,4]	0.02	0.16	0.02	0.16	0.02	0.16
	$D_{2}\left(\vec{as}\right)$		0.71	0.55	0.64	0.43	0.75	0.63
$D_{3}$	7	[5,6,6.2,7]	0.94	0.40	0.94	0.40	0.94	0.40
	8	[3,3.2,3.4,10]	0.03	0.39	0.03	0.39	0.03	0.39
	9	[1,1.4,1.6,2]	0.01	0.05	0.01	0.05	0.01	0.05
	10	[0,3.6,3.8,4]	0.02	0.16	0.02	0.16	0.02	0.16
	$D_{3}\left(\vec{as}\right)$		0.71	0.58	0.65	0.44	0.75	0.66
$as_{min}$			0.01	0.05	0.01	0.05	0.01	0.05
$as_{max}$			0.94	0.40	0.94	0.40	0.94	0.40
$w_{PARisk}$			0.5		0.9		0.3	
$w_{OOPR}$			0.5		0.1		0.7	

where *D*—aggregation method, $\vec{as}=\{as_{7},as_{8},as_{9},as_{10}\}$.

**Table 10:** The results of “full” optimization for Example 9.

D	i	${\mathrm{ar}}_{\mathrm{Fi}}\left[\%\right]$	Portfolios	
			$P_{7}$	$P_{8}$
			${\mathrm{as}}_{i}$	${\mathrm{as}}_{i}$
$D_{1}$	7	[5,6,6.2,7]	0.94	0.40
	8	[3,3.2,3.4,10]	0.03	0.39
	9	[1,1.4,1.6,2]	0.01	0.05
	10	[0,3.6,3.8,4]	0.02	0.16
	$D_{1}\left(\vec{as}\right)$		0.942	0.812
	PARisk		0.786	0.504
	OOPR		0.924	0.743
	$w_{PARisk}$		0.25	0.28
	$w_{OOPR}$		0.75	0.72
	OPR		[4.8, 5.822, 6.018, 6.98]	[3.22, 4.294, 4.462, 7.44]
$D_{2}$, $D_{3}$	7	[5,6,6.2,7]	0.94	0.40
	8	[3,3.2,3.4,10]	0.03	0.39
	9	[1,1.4,1.6,2]	0.01	0.05
	10	[0,3.6,3.8,4]	0.02	0.16
	$D_{2}\left(\vec{as}\right)$, $D_{3}\left(\vec{as}\right)$		0.924	0.742
	PARisk		0.786	0.504
	OOPR		0.924	0.743
	$w_{PARisk}$		0.0	0.0
	$w_{OOPR}$		1.0	1.0
	OPR		[4.8, 5.822, 6.018, 6.98]	[3.22, 4.294, 4.462, 7.44]
$as_{min}$			0.01	0.05
$as_{max}$			0.94	0.40

where *D*—aggregation method, $\vec{as}=\{as_{11},as_{12},as_{13},as_{14}\}$.

**Table 11:** The results of portfolio optimization (Example 10).

D	i	${\mathrm{ar}}_{\mathrm{Fi}}\left[\%\right]$	Portfolios					
			$U_{1}$	$U_{2}$	$U_{3}$	$U_{4}$	$U_{5}$	$U_{6}$
			${\mathrm{as}}_{i}$	${\mathrm{as}}_{i}$	${\mathrm{as}}_{i}$	${\mathrm{as}}_{i}$	${\mathrm{as}}_{i}$	${\mathrm{as}}_{i}$
$D_{1}$	11	[2,3,4,7]	0.02	0.06	0.02	0.06	0.01	0.06
	12	[2,5,6,7]	0.05	0.34	0.05	0.34	0.22	0.34
	13	[3,4,4.6,5]	0.04	0.08	0.04	0.08	0.03	0.08
	14	[1,4.6,6.2,6.6]	0.03	0.07	0.03	0.07	0.04	0.07
	15	[0,3,6,9]	0.01	0.05	0.01	0.05	0.02	0.05
	16	[4,4.8,5.2,6]	0.85	0.40	0.85	0.40	0.68	0.40
	$D_{1}\left(\vec{as}\right)$		0.62	0.57	0.42	0.36	0.74	0.71
$D_{2}$	11	[2,3,4,7]	0.01	0.05	0.02	0.06	0.02	0.06
	12	[2,5,6,7]	0.55	0.34	0.05	0.34	0.85	0.40
	13	[3,4,4.6,5]	0.03	0.07	0.04	0.08	0.01	0.05
	14	[1,4.6,6.2,6.6]	0.04	0.08	0.03	0.07	0.03	0.07
	15	[0,3,6,9]	0.02	0.06	0.01	0.05	0.04	0.08
	16	[4,4.8,5.2,6]	0.35	0.40	0.85	0.40	0.05	0.34
	$D_{2}\left(\vec{as}\right)$		0.49	0.47	0.40	0.35	0.58	0.55
$D_{3}$	11	[2,3,4,7]	0.01	0.05	0.02	0.06	0.02	0.06
	12	[2,5,6,7]	0.85	0.40	0.05	0.34	0.85	0.40
	13	[3,4,4.6,5]	0.02	0.06	0.03	0.07	0.01	0.05
	14	[1,4.6,6.2,6.6]	0.04	0.08	0.04	0.08	0.03	0.07
	15	[0,3,6,9]	0.03	0.07	0.01	0.05	0.05	0.15
	16	[4,4.8,5.2,6]	0.05	0.34	0.85	0.40	0.04	0.27
	$D_{3}\left(\vec{as}\right)$		0.54	0.51	0.41	0.36	0.68	0.63
$as_{min}$			0.01	0.05	0.01	0.05	0.01	0.05
$as_{max}$			0.85	0.40	0.85	0.40	0.85	0.40
$w_{PARisk}$			0.5		0.9		0.3	
$w_{OOPR}$			0.5		0.1		0.7	

where *D*—aggregation method, $\vec{as}=\{as_{11},as_{12},as_{13},as_{14},as_{15},as_{16}\}$.

**Table 12:** The result of “full’ optimization for Example 10.

D	i	${\mathrm{ar}}_{\mathrm{Fi}}\left[\%\right]$	Portfolios	
			$U_{7}$	$U_{8}$
			${\mathrm{as}}_{i}$	${\mathrm{as}}_{i}$
$D_{1}$	11	[2,3,4,7]	0.02	0.06
	12	[2,5,6,7]	0.04	0.08
	13	[3,4,4.6,5]	0.01	0.05
	14	[1,4.6,6.2,6.6]	0.05	0.34
	15	[0,3,6,9]	0.85	0.40
	16	[4,4.8,5.2,6]	0.03	0.07
	$D_{1}\left(\vec{as}\right)$		0.949	0.880
	PARisk		0.055	0.204
	OOPR		0.948	0.870
	$w_{PARisk}$		0.01	0.08
	$w_{OOPR}$		0.99	0.92
	OPR		[0.32, 3.224, 5.932, 8.63]	[1.05, 3.88, 5.822, 7.494]
$D_{2}$, $D_{3}$	11	[2,3,4,7]	0.02	0.06
	12	[2,5,6,7]	0.04	0.08
	13	[3,4,4.6,5]	0.01	0.05
	14	[1,4.6,6.2,6.6]	0.05	0.34
	15	[0,3,6,9]	0.85	0.40
	16	[4,4.8,5.2,6]	0.03	0.07
	$D_{2}\left(\vec{as}\right)$, $D_{3}\left(\vec{as}\right)$		0.948	0.870
	PARisk		0.055	0.204
	OOPR		0.948	0.870
	$w_{PARisk}$		0.0	0.0
	$w_{OOPR}$		1.0	1.0
	OPR		[0.32, 3.224, 5.932, 8.63]	[1.05, 3.88, 5.822, 7.494]
$as_{min}$			0.01	0.05
$as_{max}$			0.94	0.40

where *D*—aggregation method, $\vec{as}=\{as_{11},as_{12},as_{13},as_{14},as_{15},as_{16}\}$.

**Table 13:** The parameters of normal fuzzy assets $C_{1}-C_{5}$.

Parameters	$C_{1}$	$C_{2}$	$C_{3}$	$C_{4}$	$C_{5}$
Mean (m)	0.25	0.22	0.2	0.15	0.05
$Spread^{2}\left(w^{2}\right)$	0.0225	0.0150	0.0150	0.0100	0.0050

**Table 14:** The results of fuzzy portfolio optimization from [70].

Model	${\mathrm{as}}_{1}$	${\mathrm{as}}_{2}$	${\mathrm{as}}_{3}$	${\mathrm{as}}_{4}$	${\mathrm{as}}_{5}$
Markowitz’s model	0.185	0.194	0.212	0.237	0.171
Kataoka’s model	0.3	0.375	0.25	0.075	0
Minimum-risk model	0.425	0.375	0.2	0	0
Spread min model	0.412	0.588	0	0	0
Fractile model	0	1	0	0	0
Modality model	1	0	0	0	0
Minimax regret model	0.387	0.3	0.313	0	0

**Table 15:** The results of optimization based on the Yager’s aggregation ($D_{1}$).

No	$w_{\mathrm{OOPR}}$	$w_{\mathrm{PARisk}}$	${\mathrm{as}}_{1}$	${\mathrm{as}}_{2}$	${\mathrm{as}}_{3}$	${\mathrm{as}}_{4}$	${\mathrm{as}}_{5}$	Mean Value of Optimized
								Fuzzy Portfolio Return
1	1	0	1	0	0	0	0	0.250
2	0.95	0.05	0.97	0.03	0	0	0	0.249
3	0.75	0.25	0.86	0.14	0	0	0	0.246
4	0.5	0.5	0.68	0.32	0	0	0	0.241
5	0.35	0.65	0.54	0.46	0	0	0	0.237
6	0.2	0.8	0.36	0.64	0	0	0	0.231
7	0.1	0.9	0.2	0.8	0	0	0	0.226
8	0	1	0	1	0	0	0	0.220

**Table 16:** The results of optimization based on the multiplicative aggregation ($D_{2}$).

No	$w_{\mathrm{OOPR}}$	$w_{\mathrm{PARisk}}$	${\mathrm{as}}_{1}$	${\mathrm{as}}_{2}$	${\mathrm{as}}_{3}$	${\mathrm{as}}_{4}$	${\mathrm{as}}_{5}$	Mean Value of Optimized
								Fuzzy Portfolio Return
1	1	0	1	0	0	0	0	0.25
2	0.4	0.6	1	0	0	0	0	0.25
3	0.325	0.675	0.75	0.25	0	0	0	0.24
4	0.3	0.7	0.42	0.58	0	0	0	0.23
5	0.275	0.725	0.1	0.9	0	0	0	0.22
6	0.25	0.75	0	1	0	0	0	0.22
7	0	1	0	1	0	0	0	0.22

**Table 17:** The results of optimization based on the weighted sum aggregation ($D_{3}$).

No	$w_{\mathrm{OOPR}}$	$w_{\mathrm{PARisk}}$	${\mathrm{as}}_{1}$	${\mathrm{as}}_{2}$	${\mathrm{as}}_{3}$	${\mathrm{as}}_{4}$	${\mathrm{as}}_{5}$	Mean Value of Optimized
								Fuzzy Portfolio Return
1	1	0	1	0	0	0	0	0.25
2	0.85	0.15	1	0	0	0	0	0.25
3	0.5	0.5	1	0	0	0	0	0.25
4	0.3	0.7	0	1	0	0	0	0.22
5	0.25	0.75	0	1	0	0	0	0.22
6	0	1	0	1	0	0	0	0.22

**Table 18:** The results of optimization for Example 11.

	i	$C_{1}$	$C_{2}$	$C_{3}$	$C_{4}$	$C_{5}$	$D_{i}\left(\vec{\mathrm{as}}\right)$	$w_{\mathrm{OOPR}}$	$w_{\mathrm{PARisk}}$	OOPR	PARisk	${\mathrm{as}}_{\min}$	${\mathrm{as}}_{\max}$
		${\mathrm{as}}_{1}$	${\mathrm{as}}_{2}$	${\mathrm{as}}_{3}$	${\mathrm{as}}_{4}$	${\mathrm{as}}_{5}$							
$D_{i}$	1	0.4	0.39	0.1	0.06	0.05	0.882	0.87	0.13	0.865	0.400	0.05	0.4
	2	0.4	0.39	0.1	0.06	0.05	0.865	1	0				
	3	0.4	0.39	0.1	0.06	0.05	0.865	1	0				
	1	0.9	0.04	0.03	0.02	0.01	0.974	0.97	0.03	0.973	0.461	0.01	0.9
	2	0.9	0.04	0.03	0.02	0.01	0.973	1	0				
	3	0.9	0.04	0.03	0.02	0.01	0.973	1	0				
